# Scientific Items

**Published:** 1881-05-20

**Authors:** 


### SCIENTIFIC ITEMS.

**The Snake-Stones of Ceylon.**—The following notes are taken principally from Sir Emerson Tennent's work on Ceylon, partly also from Wood's "Natural History."

The use of the Pamboo-kaloo, or snake-stone, as a remedy in cases of wounds by venomous serpents, has probably been communicated to the Cinghalese by the itinerant snake-charmers who resort to the island from the coast of Coromandel; and more than one well-authenticated instance of its successful application has been told to Sir E. Tennent by eye-witnesses.

On one occasion, in March, 1854, some civil officers of the government were riding along a jungle path in the vicinity of Bientenne, when they saw one of two Tamils, who were approaching them, suddenly dart into the forest and return, holding in both hands a cobra de capello, which he had seized by the head and tail. He called to his companion for assistance to place it in their covered basket, but, in doing this, he handled it so inexpertly, that it seized him by the finger, and retained its hold for a few seconds, as if unable to retract its fangs. The blood flowed, and intense pain appeared to follow almost immediately; but, with all expedition, the friend of the sufferer undid his waistcloth and took from it two snake-stones, each of the size of a small almond, intensely black, and highly polished, though of an extremely light substance. These he applied, one to each wound inflicted by the teeth of the serpent. The stones attached themselves closely, the blood oozing from the bites being rapidly imbibed by the porous texture of the article applied. They adhered tenaciously for three or four minutes, the wounded man's companion in the meanwhile rubbing his arm downward from the shoulder toward the fingers. At length the snake-stones dropped off of their own accord, the suffering of the man appeared to have subsided, he twisted his fingers until the joints cracked (whether as part of the cure or in bravado Tennent does not say), and went on his way without concern.

One of these stones was sent for analysis to Professor Faraday, who pronounced it to be made of charred bone, and in all probability to have been filled with blood, and again charred. Evidence of this is afforded, as well by the apertures of cells and breaks under pressure, and exhibits an organic structure within.

Another light has been thrown on the subject by Mr. R. W. H. Hardy, who states that the snake-stone is in use in Mexico, and that it is formed by cutting a piece of stag's horn into the proper shape, wrapping it lightly in grass or hay, folding it in sheet copper so as to exclude the air, and calcining it in a charcoal fire.

**Submarine Cables.**—The first cost of submarine cables is heavy, and they last, on the average, only ten or twelve years. If a cable breaks in deep water after it is ten years old, it cannot be lifted for repairs, as it is liable to break of its own weight. The action of the sea water gradually destroys the outer coating of iron wire, though the core of the cable may remain perfect. The companies are consequently compelled to put aside a large share of their earnings as a reserved fund for this decennial renewing of the cables.

The repairs of these submarine lines are also very costly. A ship has to be chartered at an expense of some five hundred dollars a day; and it generally takes several weeks to find the locality of the break and to mend it, which can be done only in favorable weather. A single break has sometimes cost a hundred thousand dollars.

Still, this branch of telegraphy is profitable, and new lines are constantly being laid. There are six wires connecting this country with Great Britain and France, and it is announced that two more will soon be added.—*Journal of Chemistry*.

**Cold Weather and Health.**—In his late report as registrar of Providence, Dr. Snow remarks: "There is a popular error, which we often hear spoken of in the winter season, that clear, cold weather is favorable to the public health. The truth is that *in this climate* severe cold weather, if continued more than two or three days, increases the number of deaths as certainly as continued hot weather, though in a different manner. Severe cold depresses the vital forces, and exposure to it produces fatal results among those persons, or classes of persons, whose vital force is weakened by any cause. Such persons are the aged and the very young, and also all who are sick or debilitated from any other cause. Besides this, severe cold is no preventive of, but on the contrary is favorable to, the spread of some of our most fatal diseases, as small-pox, diphtheria, and scarlitina. This is shown at the present time in Brooklyn, New York, Philadelphia, Chicago, and other places.—*Ibid.*

**A New Test of Intelligence.**—The Parisian scientist, Dr. Delaunay, has made the curious discovery that, to ascertain the qualities of a cook, it is sufflcient to give her a plate to clean, or sauce to make, and watch how she moves her hand in either act. If she move it from left to right, or in the direction of the hands of a watch, you may trust her; if the other way, she is certain to be stupid and incapable. Similarly, the intelligence of people may be gauged by asking them to make a circle on paper with a pencil, and noting in which direction the hand is moved. The good students, in a mathematical class, draw circles from left to right. The inferiority of the softer sex (as well as of male dunces) is shown by their drawing from right to left; asylum patients and children do the same. In a word, *centrifugal* movements are a characteristic of intelligence and higher development; *centripetal* are a mark of incomplete evolution. A person, as his faculties are developed, may come to draw circles the opposite way to what he did in youth. Dr. Delaunay has some further extraordinary conclusions as to the relative positions of races in the scale of development from the way they wind their watches and make their screws.—*Ibid.*

